# A novel signature incorporating genes related to lipid metabolism and immune for prognostic and functional prediction of breast cancer

**DOI:** 10.18632/aging.205828

**Published:** 2024-05-20

**Authors:** Xiao Zhao, Lvjun Yan, Zailin Yang, Hui Zhang, Lingshuang Kong, Na Zhang, Yongpeng He

**Affiliations:** 1 Clinical Laboratory, People’s Hospital of Xinjin District, Chengdu 611430, China; 2 Chongqing Key Laboratory of Translational Research for Cancer Metastasis and Individualized Treatment, Chongqing University Cancer Hospital and Chongqing Cancer Institute and Chongqing Cancer Hospital, Chongqing 400030, China; 3 Tumor and Hematology Department, University-Town Hospital of Chongqing Medical University, Chongqing 401331, China

**Keywords:** lipid metabolism, immunity, breast cancer, IL18, prognosis, RT-PCR

## Abstract

Purpose: Breast cancer prognosis and functioning have not been thoroughly examined in relation to immunological and lipid metabolism. However, there is a lack of prognostic and functional analyses of the relationship between lipid metabolism and immunity in breast cancer.

Methods: DEGs in breast cancer were obtained from UCSC database, and lipid metabolism and immune-related genes were obtained from GSEA and Immune databases. A predictive signature was constructed using univariate Cox and LASSO regression on lipid metabolism and immune-related DEGs. The signature’s prognostic significance was assessed using Kaplan-Meier, time-dependent ROC, and risk factor survival scores. Survival prognosis, therapeutic relevance, and functional enrichment were used to mine model gene biology. We selected IL18, which has never been reported in breast cancer before, in the signature to learn more about its function, potential to predict outcome, and immune system role. RT-PCR was performed to verify the true expression level of IL18.

Results: A total of 136 DEGs associated with breast cancer responses to both immunity and lipid metabolism. Nine key genes (CALR, CCL5, CEPT, FTT3, CXCL13, FLT3, IL12B, IL18, and IL24, *p* < 1.6e^−2^) of breast cancer were identified, and a prognostic was successfully constructed with a good predictive ability. IL18 in the model also had good clinical prognostic guidance value and immune regulation and therapeutic potential. Furthermore, the expression of IL18 was higher than that in paracancerous tissue.

Conclusions: A unique predictive signature model could effectively predict the prognosis of breast cancer, which can not only achieve survival prediction, but also screen out key genes with important functional mechanisms to guide clinical drug experiments.

## INTRODUCTION

In recent years, breast cancer has eclipsed lung cancer as a major health threat to women. Malignant breast cancers form in the ductal and lobular areas [1, 2]. Recently, more than 20% of newly diagnosed cancers in Chinese women are breast cancer [3]. The primary clinical intervention among them continues to be pharmacological therapy, and with the introduction of immunomodulatory drug therapy, the treatment resources have significantly improved. However, during treatment, a sizable portion of breast cancer patients display treatment resistance and post-treatment recurrence [4]. As a consequence, a detailed understanding of breast cancer pathophysiology and survival markers is needed to improve postoperative treatment and patient survival [5].

Metabolic reprogramming is a characteristic of malignant cell development, according to the “Warburg effect” [6, 7] Lipids, which are made up of vital components such as fatty acids, glycerophospholipids, and sphingolipids, play an important function in the human body [8]. A growing number of studies show that lipid metabolism is involved in a variety of processes, ranging from tumor cell genesis to apoptosis. Phosphatidic acid and sphingolipids, for example, can operate as second messengers and play roles in cell differentiation, apoptosis, and cell cycle arrest [9, 10]. According to a clinical data meta-analysis [11], the risk of lung cancer is frequently associated with a rise in total cholesterol and a decrease in triglycerides in patients. Breast cancer cells contain a range of membrane lipids, and higher levels of endogenous fatty acids, such as palmitate-containing phosphatidylcholine, have been linked to tumor development and survival [12]. In the meantime, a further meta-analysis revealed that dietary dysregulation of the cholesterol pathway is linked to an increased risk of breast cancer [13]. Breast cancer cells also absorb extra cholesterol, which promotes cancer cell growth and migration [14, 15]. Immunotherapy has increased breast cancer survival; therefore, changes in the tumor immune microenvironment affect post-operative treatment. Lipid metabolism genes regulate tumor immune systems and prognosis. Thus, lipid metabolism pathway markers should be examined in relation to immune function and breast cancer prognosis.

On this basis, a novel feature model with good predictive ability for breast cancer was constructed. After comparing several independent factors, the biomarker IL18 was screened out with a variety of important research value. In view of the results, we firmly believe that IL18 and the other related genes in this study have significant potential as prognostic value. Besides, this work offers novel immunotherapy and breast cancer prognostic insights.

## MATERIALS AND METHODS

### Data collection and preprocessing

Breast cancer and control data were obtained from the UCSC public database (https://xenabrowser.net). The DesSeq2 package was used to evaluate differential gene expression in 1072 breast cancer and 99 paraneoplastic control samples. Genes with adjusted *P* < 0.05 and |logFC| ≥1 differential thresholds were filtered (Supplementary Table 1). GSEA (https://www.gsea-msigdb.org) and the ImmPort database (https://www.immport.org) were consulted for obtaining the immune pathway gene set and lipid metabolism gene set [16, 17] (Supplementary Table 2). The datasets were then analyzed using the Venn diagram website to identify overlapping genes, yielding 136 DEGs associated with breast cancer responses to both immunity and lipid metabolism (http://bioinfogp.cnb.csic.es/tools/venny/index.html). Details of the genes associated with lipid metabolism and immunity are shown in Supplementary Table 3. In addition, the “pheatmap” package was used to construct a heatmap. This shows how overlapping genes express differently.

### Functional annotation and enhancement analyses

For 136 genes that intersected, we carried out KEGG pathway analysis and GO enrichment analysis. The “enrichplot” and “clusterProfiler” programs were used in this work to produce KEGG scatter plots and GO enrichment scatter plots of genes that interacted.

### Prognostic modeling

The DEGs associated with breast cancer OS were selected using univariate Cox regression. Using LASSO regression, DEGs associated with OS of breast cancer were selected. We used the R tool ‘glmnet’ to identify independent prognostic markers (*P* < 0.05). The UCSC dataset was sorted 1:1 into training and test cohorts to assess independent factors affecting OS. The formula for the risk score obtained according to the LASSO regression results was as follows:


$$\text{risk}\;\text{score}=\sum_{i=1}^{n}\left({\text{Coef}}_{i}\times {\text{Exp}}_{\text{i}}\right)$$

Where *n* is the number of prognosis-related genes in the model, Coefi the related gene coefficient, and Expi represents gene expression. High-risk and low-risk groups were identified for the training and test sets, and the survival coefficient versus the ROC curve (AUC value) indicated the model’s prognostic value. The ‘forestplot’ tool created a forest plot to show the candidate genes’ HR scores (*P* < 0.05).

### Gene expression pattern study

The candidate genes’ PPI protein enrichment network was analyzed after investigating the model genes’ upstream and downstream interactions using the STRING database (https://string-db.org). Then, the UALCAN database (https://ualcan.path.uab.edu) was used to compare candidate gene mRNA expression in breast cancer’s primary site and normal tissues, as well as protein expression levels [18]. SWISS-Model (https://swissmodel.expasy.org) also exhibited the protein 3D structural prediction model of the candidate markers. In addition to demonstrating the protein expression pattern of candidate genes through the HPA (https://www.proteinatlas.org) database, we tapped the subcellular localization results of target genes to demonstrate the protein exercise functional regions [19].

### Gene function analysis

The ROC curves of single genes in the candidate gene set were plotted, and AUC values were calculated through an online website (http://bioinformatics.com.cn). In addition, the GEPIA2 website (http://gepia2.cancer-pku.cn) was utilized to mine the OS survival curves of the candidate genes. And the OS survival coefficients of key genes in pan-cancer species were analyzed in conjunction with the Kaplan-Meier Plotter database (http://kmplot.com), and only those cancer species with a significance level of *P* < 0.05 were demonstrated. Meanwhile, in order to explore the DNA methylation levels and functional mechanisms of target genes in breast cancer patients, the information on CpG islands of DNA methylation sites and the correlation with survival prognosis were mined online through the MEXPRESS website (https://mexpress.ugent.be/) [20]. Finally, combined with CancerSEA (http://biocc.hrbmu.edu.cn/CancerSEA/home.jsp) to decode pan-cancer species functional information of key genes at a single-cell resolution level [21].

### Correlation between gene expression levels and immune mechanisms

The TIMERs algorithm (http://timer.cistrome.org) was used to visualize and demonstrate Spearman’s correlation between immune infiltration estimates and gene expression [22]. In addition, the transcript levels of target genes were analyzed in conjunction with the TISIDB database (http://cis.hku.hk/TISIDB/index.php) to analyze the enrichment levels of immune subtype functions in different tumor microenvironments: C1 (wound healing); C2 (IFN-gamma dominant); C3 (inflammatory); C4 (lymphocyte depleted); C5 (immunologically quiet); and C6 (TGF-b dominant). Meanwhile, the RNA transcriptome expression level comparison was selected through LinkedOmics (https://www.linkedomics.org/admin.php) to screen the set of genes positively or negatively regulated by the target genes as well as the enriched pathways.

### Drug sensitivity analysis

In order to explore the target drug information of potential genes, this project mined drug information with Spearman correlation with candidate gene expression in the cancer database (GDSC, https://www.cancerrxgene.org/). By combining the GDSC and CTRP tumor cell line drug databases, we will explore drugs with positive and negative regulatory mechanisms and correlations with gene expression levels.

### Single-cell database mining of target genes

In order to explore the expression patterns of target proteins at the single-cell resolution level, this study mined the expression patterns and sites of candidate genes in a number of breast cancer single-cell datasets by using the single-cell database TISCH (http://tisch.comp-genomics.org), as well as evaluating the risk coefficients of the target genes in pan-cancer species by combining them with the TCGA database (*P* < 0.05). The interaction factors of target genes were also mined in conjunction with single-cell datasets. Meanwhile, t-SNE downscaling analysis and expression sites of target genes in the mammary tissues of the model animal mice were mined online by the Tabula Muris database (https://tabula-muris.ds.czbiohub.org).

### Human specimens

Three pairs of breast cancer and paracancerous specimens were collected, which were obtained from clinical postoperative tissue. After specimen isolation, tissue was frozen rapidly in liquid nitrogen and stored in a refrigerator at −80°C to prevent degradation.

### RT-PCR

Tissue samples were extracted from Trizol reagent (Invitrogen, Carlsbad, CA, USA) and reverse transcribed to mRNA. The primers are as follows: L18-F: GATAGCCAGCCTAGAGGTATGG; IL18-R: CCTTGATGTTATCAGGAGGATTCA; ACTIN-F: CACCATTGGCAATGAGCGGTTC; ACTIN-R: AGGTCTTTGCGGATGTCCACGT. Samples are tested in biological replicates (number of replicates = 3 replicates).

### Statistical analyses

R software 4.2.0 was used for data analysis and visualization. Comparisons among both groups were made with the Wilcoxon rank-sum test, and comparisons between two or more groups were implemented through the Kruskal-Wallis test. Comparisons of categorical variables were conducted utilizing the chi-square test or Fisher’s exact test. The differences between survival curves were identified via applying the log-rank test. Associations between both variables were evaluated with Spearman’s correlation test. Statistical significance was set to *P* < 0.05 significant.

### Data availability

The datasets generated during and/or analyzed during the current study are available from the corresponding author on reasonable request.

## RESULTS

### Acquisition of genes related to lipid metabolism and immunology

**Figure 1:**
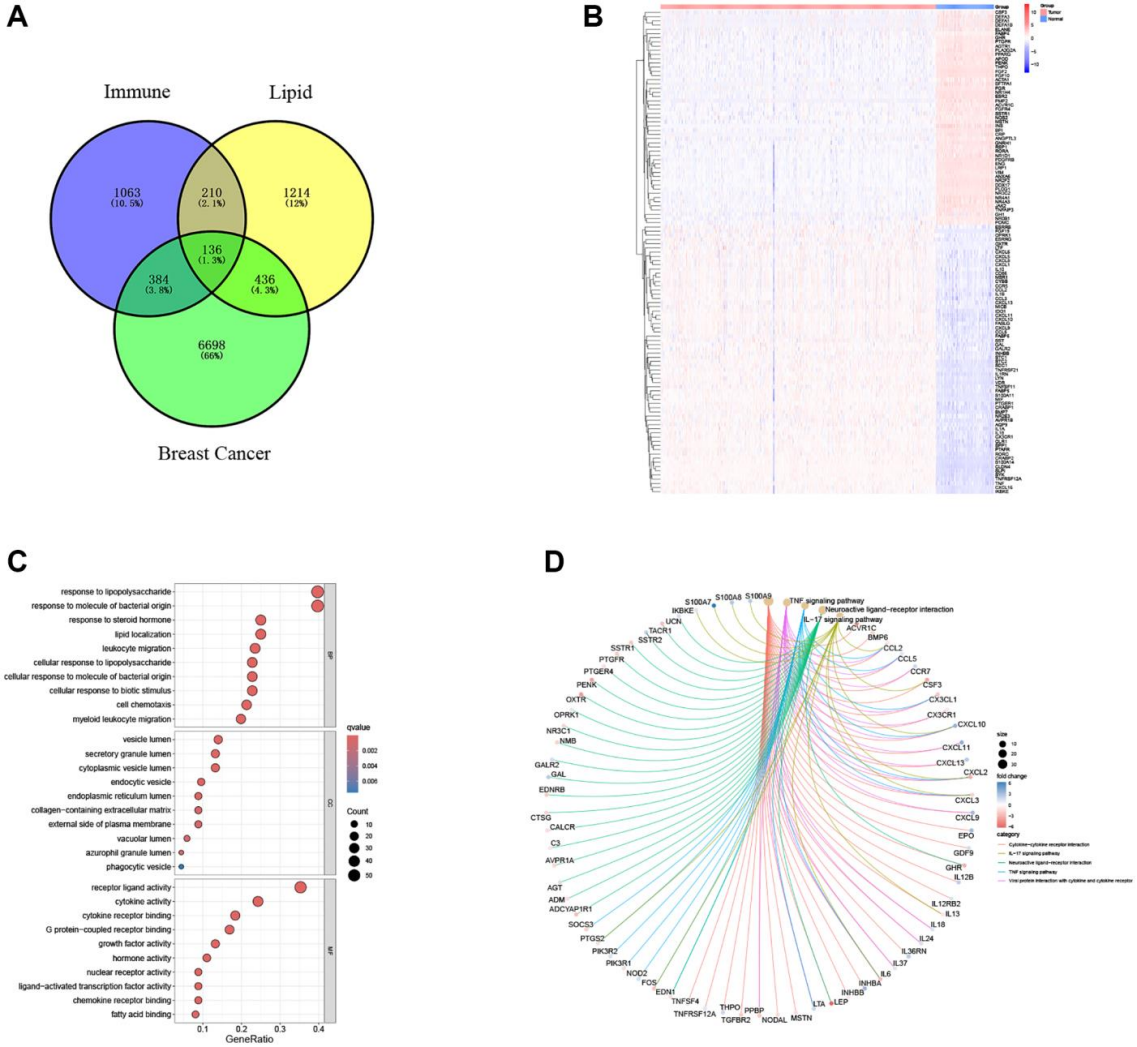
**Exploration of immune-related and lipid metabolism-related DEGs.** (**A**) Venn diagram shows the intersection of lipid metabolism and immune pathway gene sets with breast cancer DEGs. (**B**) The heatmap showed the difference of intersection gene expression level. (**C**) Gene ontology (GO) functional annotation. (**D**) KEGG pathway enrichment analysis of captured gene sets.

A total of 7654 genes with substantial expression differences were identified. Next, 136 genes with co-intersecting sets were screened through the Venn diagram analysis website (Figure 1A), and a heat map was drawn based on the expression differences of the gene sets (Figure 1B). The ‘clusterProfiler’ R package (Figure 1C, 1D) was used to calculate GO functional enrichment and KEGG pathway results to explore the functional effects of intersecting gene maps. GO and KEGG analyses showed that the genes involved in both lipid metabolism and immunity may be involved in the regulation of lipopolysaccharide and steroid hormone metabolism, as well as changes in cytokine function and the TNF signaling pathway in breast cancer patients.

### Prognostic marker exploration

**Figure 2:**
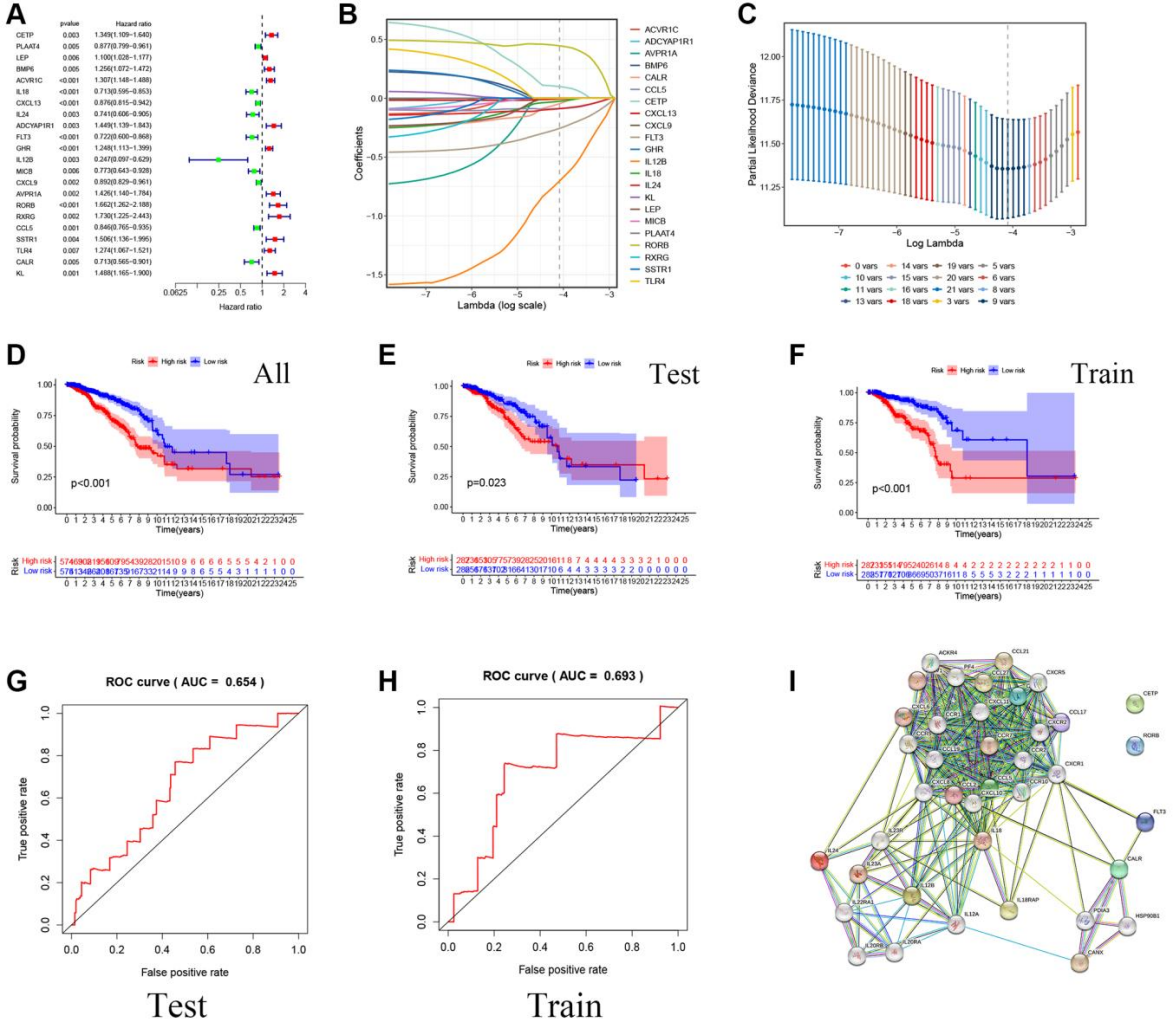
**Construction of a prognostic model for Breast cancer patients based on immune-related and lipid metabolism-related DEGs.** (**A**) The forest map shows the results of single factor analysis. (**B**–**C**) LASSO coefficient profile analysis and cross-validation to identify the most useful prognostic genes. (**D**–**F**) Kaplan-Meier curves of OS in the TCGA cohort based on risk score. (**G**–**H**) The time-dependent ROC curves for the prognostic signature base on risk score. (**I**) Construction of Prognostic gene PPI network based on STRING database.

The univariate Cox analysis was performed on 136 genes, and a total of 22 genes related to survival selection were identified (significance *p* < 0.01), and the HR level was demonstrated with a forest plot (Figure 2A). Immediately after, nine prognostic factors with independent influences were filtered out using LASSO and Cox (Figure 2B, 2C). Modelled risk score calculations were carried out to categorize all breast cancer data into low-risk and high-risk groups according to the median score. In addition, the TCGA dataset was randomly divided into training and validation sets in a 5:5 ratio to verify the stability of the model from multiple perspectives. Plotting of K-M survival curves showed that the total data set, training set, as well as test set showed that the high-risk group had a worse prognosis than the low-risk group (significance *p* < 0.05) (Figure 2D–2F). Meanwhile, ROC curve results showed that the model has a prognostic value (Figure 2G, 2H). At the same time, the nine model screening genes were put through the PPI protein interaction network diagram to show the upstream and downstream protein interaction information, and it can be found that IL18, CXCL13, and so on have rich communication response mechanisms (Figure 2I).

### Prognostic marker information mining

**Figure 3:**
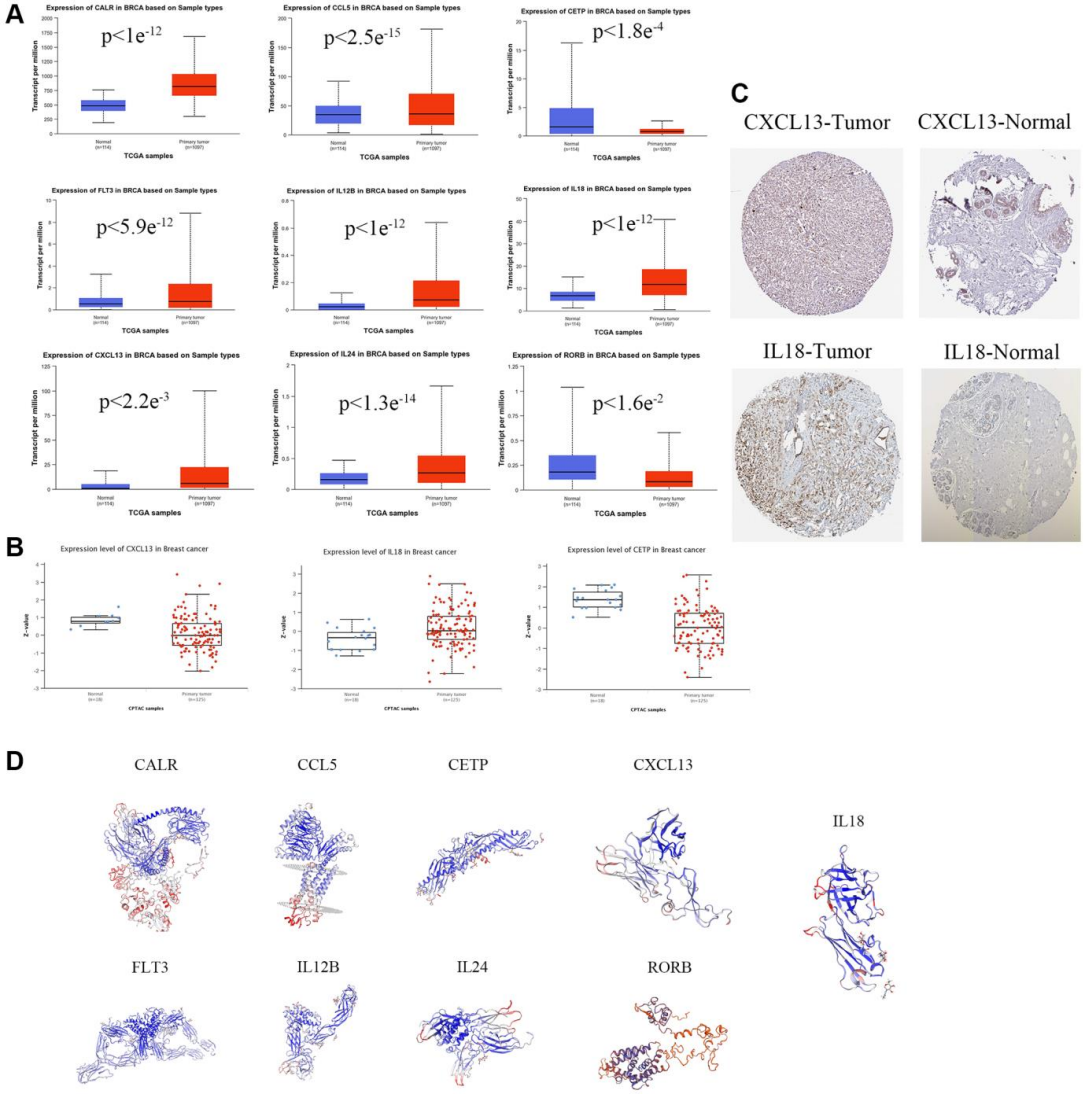
**Prognostic gene expression levels in Breast cancers.** (**A**) mRNA expression levels of prognostic genes. (**B**) The expression level of prognostic protein was based on CTPAC database. (**C**) Representative immunohistochemical staining images of prognostic gene (https://v17.proteinatlas.org/images/52613/126777_A_4_2.jpg. https://v17.proteinatlas.org/images/52613/126780_B_2_4.jpg. https://v17.proteinatlas.org/images/7772/19616_A_4_7.jpg. https://v17.proteinatlas.org/images/3980/13571_B_1_4.jpg). (**D**) The protein 3D spatial structure of prognostic factors.

In order to visualize the expression of single prognostic markers, in this study, the transcriptional data of nine prognostic gene mRNAs were studied by the UALCAN database(https://ualcan.path.uab.edu), in which the transcript levels of CALR, CCL5, CXCL13, FLT3, IL12B, IL18, and IL24 were higher than those of normal tissues in the primary tumor tissues of breast cancer patients *in vivo* (Figure 3A). In addition, the information of genes with protein data was mined by the protein expression database (CPTAC) (https://www.proteinatlas.org), in which the protein levels of CETP, CXCL13, and IL18 were expressed at the same level as mRNA (Figure 3B). In addition, immunohistochemistry results of tumor sections and normal tissue sections showed differential protein expression of CXCL13, and IL18 (Figure 3C) (https://v17.proteinatlas.org/images/52613/126777_A_4_2.jpg. https://v17.proteinatlas.org/images/52613/‌126780_B_2_4.jpg. https://v17.proteinatlas.org/images/‌7772/19616_A_4_7.jpg. https://v17.proteinatlas.org/‌images/3980/13571_B_1_4.jpg). And the protein 3D predicted structures of prognostic markers were mined by SWISS-Model online database (https://swissmodel.expasy.org), and the results showed that the protein structures of breast cancer prognostic markers all had complex folding and spatial structures (Figure 3D).

### Prognostic mechanism and drug sensitivity of model genes

**Figure 4:**
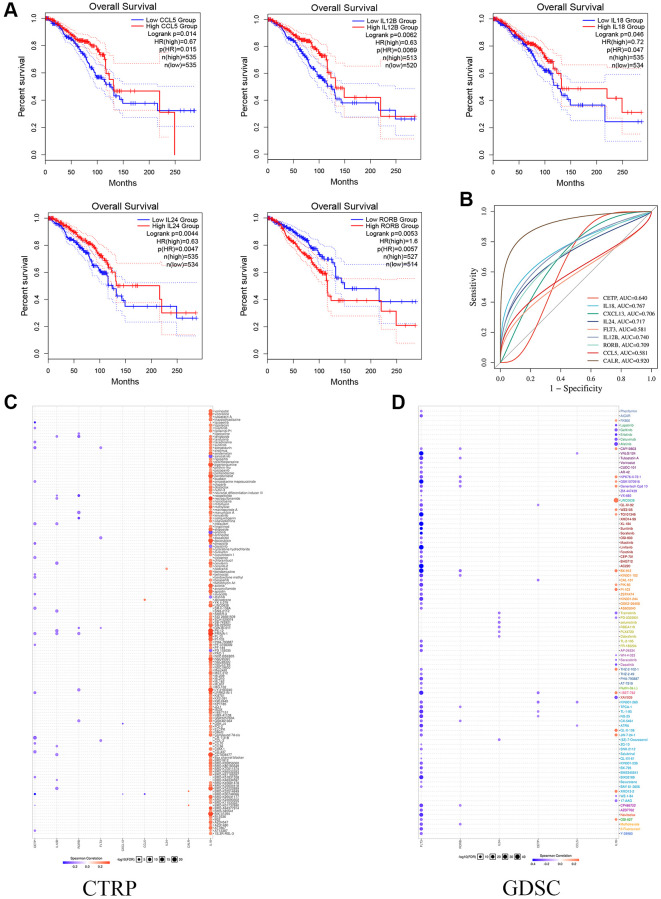
**The Functional effects of prognostic genes.** (**A**) Kaplan-Meier curves show the survival of single gene OS in prognostic model. (**B**) The ROC analysis of model gene. (**C**, **D**) The CTRP and CDSC databases show potential targets for prognostic genes.

This study analyzed the link between OS and 9 prognostic markers using the GEPIA database. We identified 5 genes with a significant correlation with OS survival prognosis using logrank *P* < 0.05 (Figure 4A). On the ROC analysis website (https://www.bioinformatics.com.cn), we examined the AUC values of the genes contributing to the model. CALR and IL18 had AUCs above 0.75, indicating that the single index genes in the model had good clinical prognostic guidance value (Figure 4B).

In addition, to explore the correlation between expression level genes and drug sensitivity (IC50 value) of potential small-molecule compounds. First, the CTRP cell line library results showed that all 9 genes had potential correlation for targeting small-molecule compounds, and notably, the expression of the IL18 gene responded to enhanced drug sensitivity (Figure 4C). Finally, the GDSC cell line correlation results similarly showed that the expression levels of FLT3 and IL18 were correlative to multiple small-molecule compound drug sensitivities (Figure 4D). Notably, the results from both databases suggest that the breast cancer IL18 gene functions as an important drug target.

### Expression levels and methylation profiles of key candidate genes

**Figure 5:**
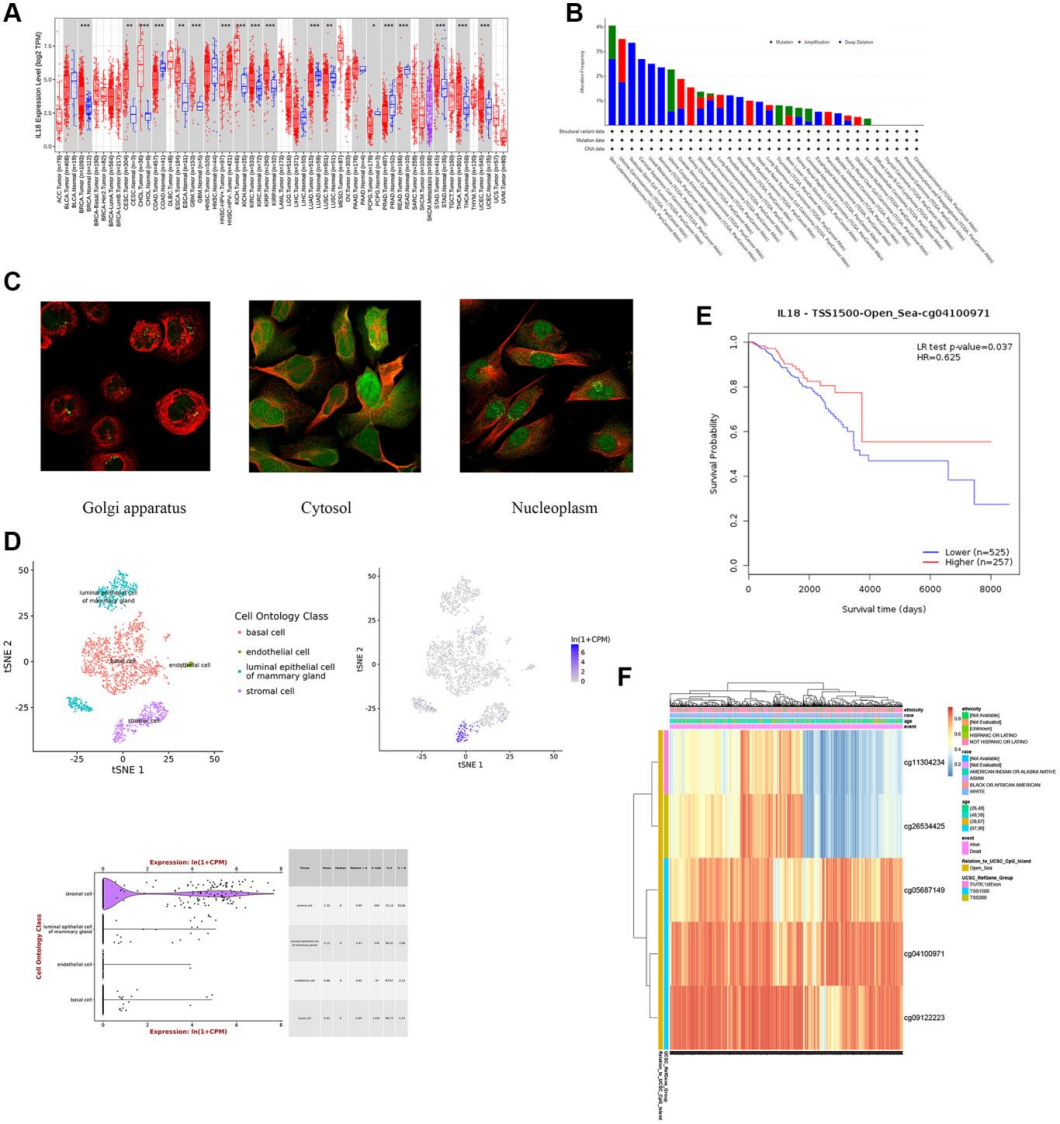
**The analysis of biological effects of key gene -IL18.** (**A**) IL18 expression in different cancers and paired normal tissue in the TIMER2 database. (**B**) IL18 gene mutation types in different cancer species. (**C**) Subcellular localization of IL18 gene in different cancer cell lines. (**D**) The expression information of IL18 at single cell level was analyzed in model animal mouse single cell database. (**E**, **F**) DNA methylation database showed that methylation levels at different sites of IL18 were correlated with survival and prognosis.

Based on the TCGA database analysis, the IL18 expression levels were significantly higher in 11 cancer species than in paracancerous tissues, including BRCA, CESC, CHOL, ESCA, GBM, KICH, KIRC, KIRP, STAD, THCA, and UCEC (Figure 5A). Meanwhile, the cBioPortal database demonstrated genomic mutation information of the key gene IL18 in different malignant tumor tissues (Figure 5B). In addition, this study obtained subcellular localization information for IL18 from the A-431, U-251MG and U-20S osteosarcoma cell lines in the HPA database. Immunofluorescence results demonstrated that the IL18 protein could be expressed in multiple regions, such as the Golgi apparatus, Nucleoplasm, and Cytosol (Figure 5C). Meanwhile, an unbiased clustering method combined with IL18 expression level was used for cell clustering and t-SNE dimensionality reduction analysis of mouse mammary gland cells, and the results showed that IL18 expression was mainly in the stromal cell in mouse mammary gland tissues (Figure 5D).

In addition, this study also profiled IL18 methylation levels, CpG site data, and CpG island associations with predictive value using a DNA methylation database. The K-M curve of cg0410097 of TSS1500 of IL18 demonstrated that increased methylation may promote breast cancer survival (Figure 5E, 5F). Heatmaps showed IL18’s methylated CpG sites (cg09122223, cg04100971, cg05687149, cg26534425, cg11304234). The results confirmed that IL18 methylation influences breast cancer survival.

### Correlation between IL18 and immune mechanism

**Figure 6:**
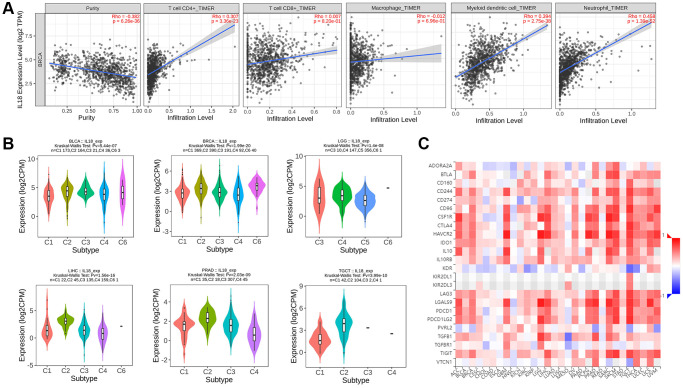
**The immune mechanism of IL18.** (**A**) The correlation between IL18 expression level and immune cell infiltration was calculated based on TIMER2.0 algorithm. (**B**) The correlation between IL18 expression and immune subtypes in different cancers. (**C**) Correlation analysis between il-18 and immune checkpoint inhibitor sites.

Since this study is based on the key genes of the immune pathway screen, we tried to apply the TIMER2 algorithm to resolve the association between IL18 expression level and immune cell infiltration in the TME. The results pointed out that IL18 expression level might be associated with T cell CD4+, neutrophil, and myeloid dendritic cell (DC cell) infiltration, and there was no significant correlation with the level of T cell CD8+ and macrophage infiltration (*P* > 0.05) (Figure 6A). Then, the distribution of IL18 enrichment in six Immune subtypes in pan-cancer species was explored on the Tumor and Immune System Interactions Online website: C1 (wound healing), C2 (IFN-γ dominance), C3 (inflammation), C4 (lymphocyte depletion), C5 (immunologically quiet), and C6 (TGF-b dominance). The cancer types with high Kruskal-Wallis Test (-log10pv) values in the pan-cancer species histogram were selected, in which BLCA and BRCA had the same expression pattern, both expressing C1, C2, C3, C4, and C6 (Figure 6B). And the correlation analysis of IL18 with immune checkpoint inhibitors at the pan-cancer level showed that the high expression of IL18 levels in breast cancer patients might be related to CD244, CTLA4, HACVR2, etc. (Figure 6C). The results of the rich immune pathway analysis suggest that IL18 in cancer may be involved in mobilizing the tumor immune microenvironment response and the assessment of the efficacy of immunosuppressants and other drugs.

### Prognostic mechanisms of the IL18 in pan-cancer species

**Figure 7:**
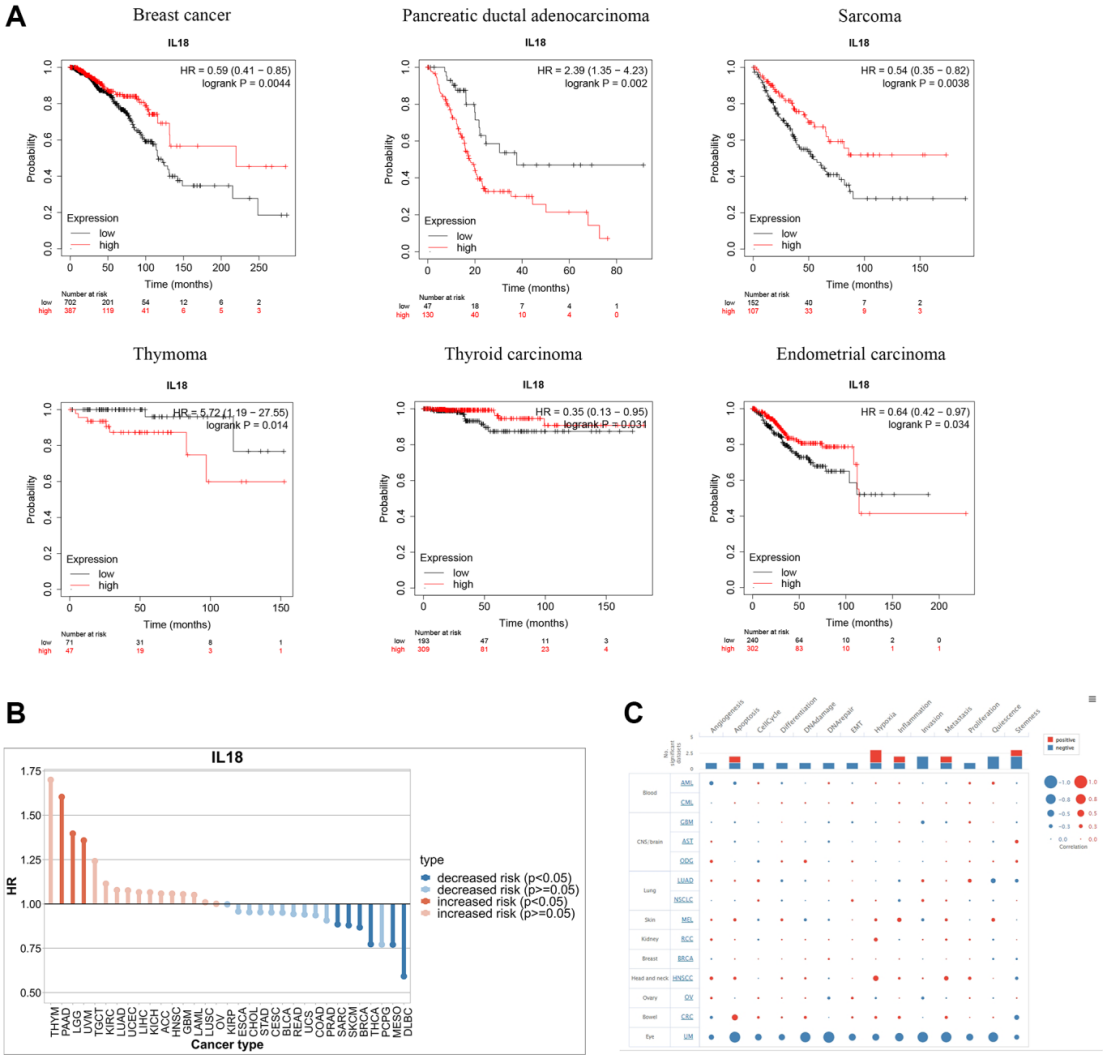
**Functional enrichment analysis of IL18 in Pan-cancer.** (**A**) Kaplan-Meier curves show the OS survival of IL18 in Pan-cancer. (**B**) The difference in prognostic risk for IL18 in Pan-cancer. (**C**) The bubble map reveals the biological enrichment of IL18 in Pan-cancer.

Based on 21 tumors from the Kaplan-Meier Plotter website, the prognostic correlation of the IL18 expression levels in different cancer types was explored. K-M survival curves indicated that, among them, breast cancer, sarcoma, pancreatic ductal adenocarcinoma, thymoma, thyroid carcinoma, uterine corpus endometrial carcinoma, The IL18 expression level had a significant correlation with disease prognosis (*p* < 0.05) (Figure 7A). Meanwhile, drawing on the survival information of the TCGA database to explore the survival prognostic value of IL18 in pan-cancer, it could be found that the high expression of the IL18 reduced the risk of breast cancer progression (*p* < 0.05), and it was noteworthy that the high expression of IL18 increased the survival risk of PAAD, LGG, and UVM (*p* < 0.05) (Figure 7B). Then, we mined the functional differences of IL18 in multiple cancer species at single-cell resolution in the CancerSEA database. The results showed that the report of IL18 in breast cancer was still in the early stages, but the bubble map distribution could reveal that IL18 was involved in several mechanisms, such as apoptosis, DNA repair, DNA damage, metastasis, and so on, in patients with uveal melanoma (Figure 7C). In summary, it can be found that the IL18 expression not only regulates the survival and prognosis of many types of cancers but also has different functions in different cancer species.

### Biological effects of the IL18 at the single-cell level

**Figure 8:**
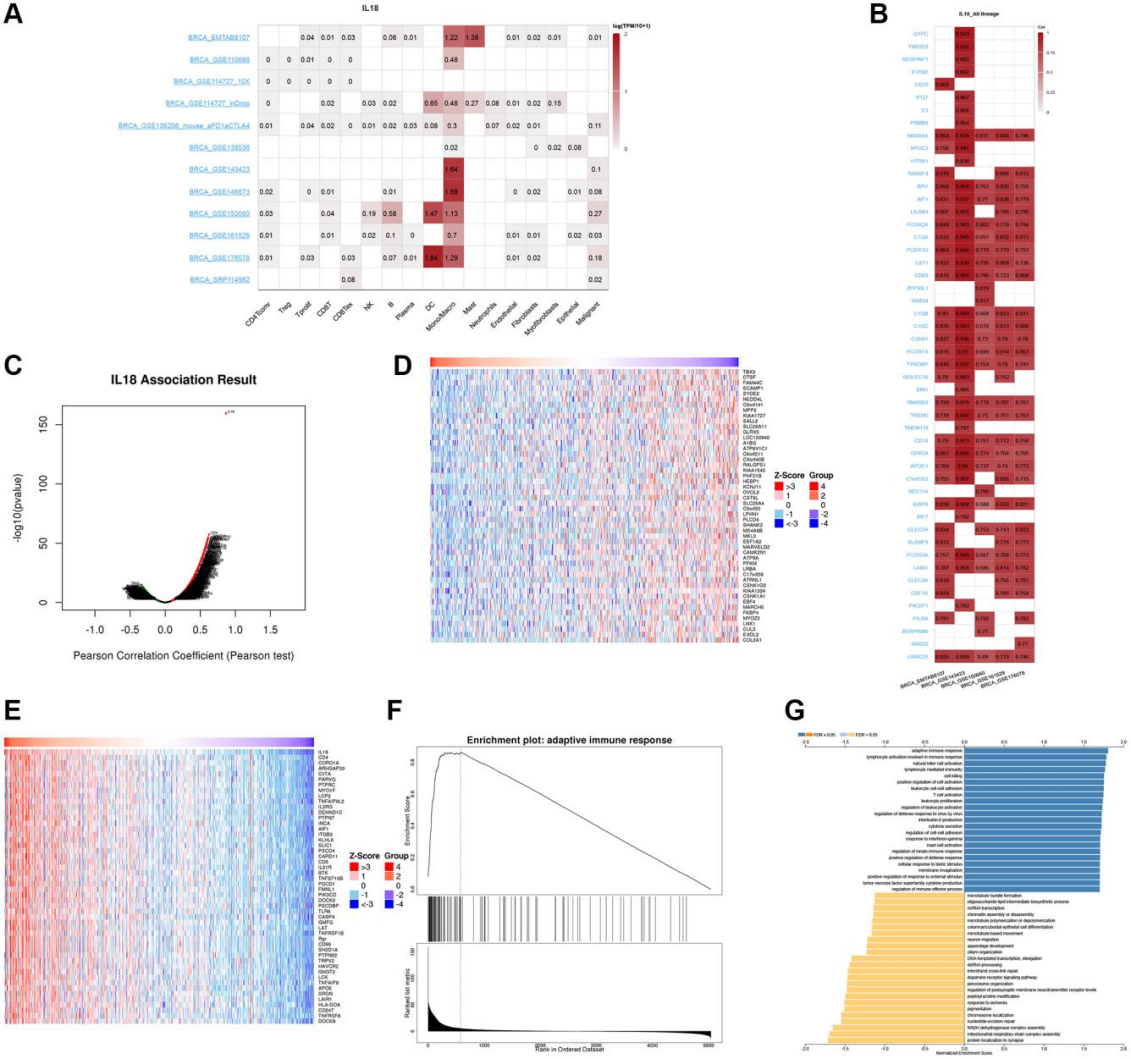
**The Single cell database analysis of IL18.** (**A**, **B**) Single cell sequencing data from different breast cancers showed the expression of IL18 enriched cells and interacting genes. (**C**–**E**) The LinkedOmics database shows the IL18 up-regulated and down-regulated gene sets. (**F**, **G**) Functional effects of IL18 interacting genes were analyzed by GSEA and GO enrichment.

The TISCH2 database was used to get information about the link between IL18 levels in breast cancer cells and the area around the tumor. The findings from different datasets revealed that the IL18 was mainly responsive in myeloid dendritic cells (DC cells) and monocyte and macrophage cells (Figure 8A). Simultaneous analysis of the EMTAB8107, GSE143423, GSE150660, GSE161529, and GSE176078 datasets demonstrated interactions between proteins of IL18. Several sets of data showed at the same time that the genes SPI1, AIF1, LILRB4, FCGR2A, C1QA, FCER1G, LST1, and CD68 were strongly linked to IL18 (Figure 8B).

### Functional enrichment of IL18

To investigate the potential molecular mechanisms of the IL18 in cancer, we established the functional enrichment of IL18 through the LinkedOmics website, and the volcano plot demonstrated the results of gene enrichment of the regulatory network associated with IL18 (Figure 8C), in which the heatmap demonstrated the sets of differentially expressed genes positively and negatively regulated by IL18 (Figure 8D, 8E). Enrichment of IL18 interaction network genes revealed that the IL18 regulatory network mainly functions in response to adaptive immune response, lymphocyte activation involved in immune response, and natural killer cell activation.

**Figure 9:**
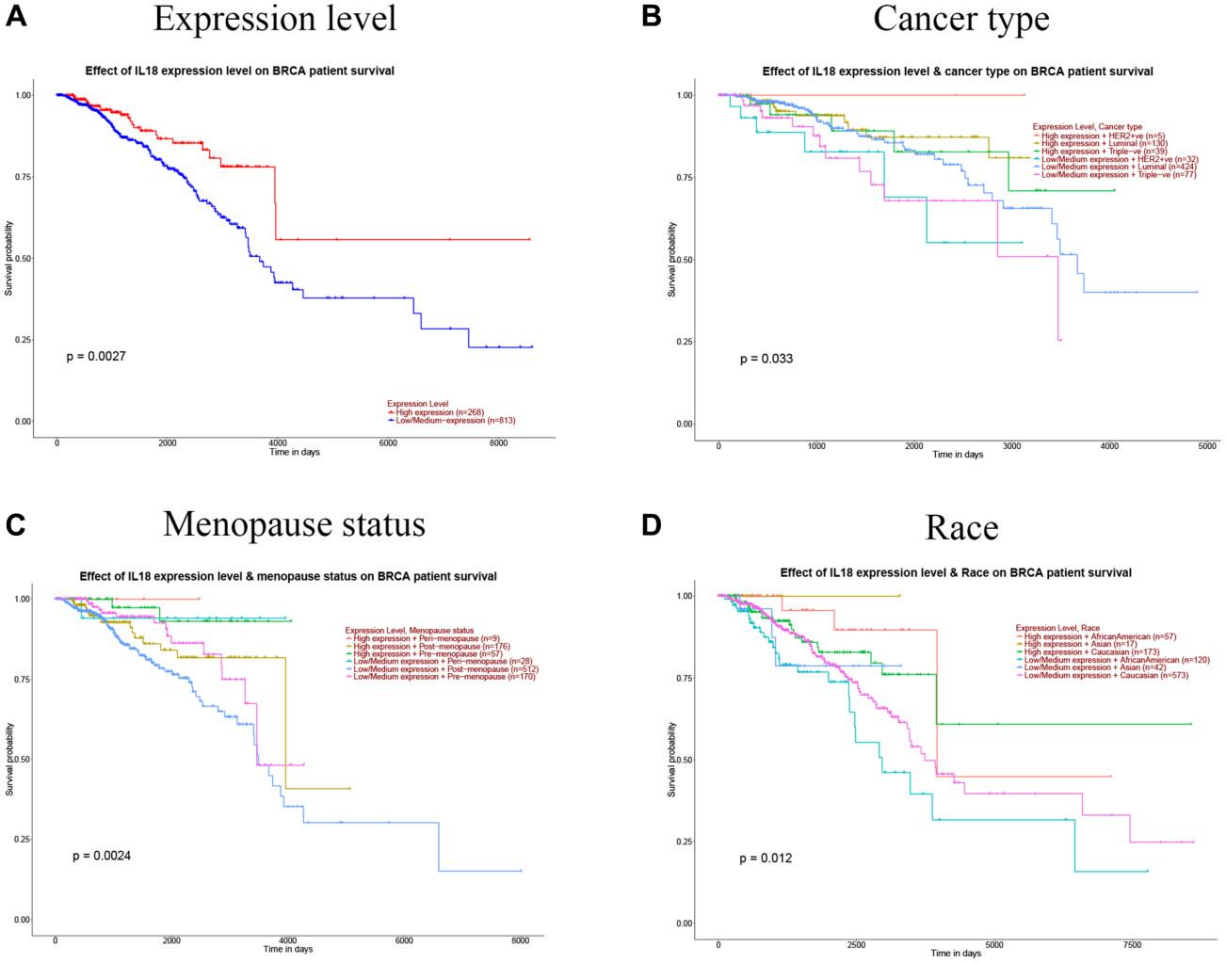
**Correlation between IL18 expression levels and clinical information.** (**A**) IL18 expression level and breast cancer survival; (**B**) IL18 expression level and breast cancer type; (**C**) Correlation of IL18 expression level with menopausal status of breast cancer patients. (**D**) The correlation between the expression level of 8 and race was analyzed. (Significant designations are: *P* < 0.01).

When GSEA was used to find the most important pathways, it mostly found GO 0002250: adaptive immune response pathway (Figure. 8F, 8G). We also found that IL18 gene expression was significantly correlated with survival, different breast cancer subtypes, race and menopausal status using the TCGA public database (*P* < 0.05). (Figure 9A–9D). In summary, the IL18 gene is not only involved in multiple clinical indications of breast cancer, but also participates in the regulation of immune response pathways through interacting genes.

### Expression of IL18 in breast cancer and adjacent tissues

**Figure 10:**
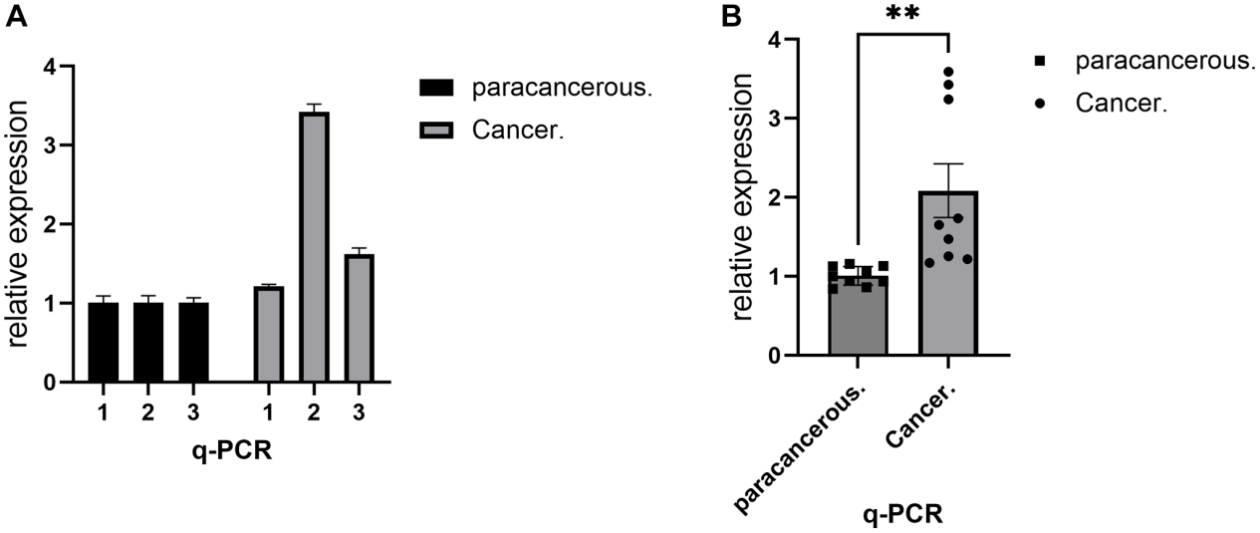
**RT-PCR demonstrated the expression level of IL18 in breast cancer tissues.** (**A**) RT-PCR assay was used to show the difference in IL18 transcript levels in a single sample between the experimental and control groups. (**B**) RT-PCR experiments showing group differences in IL18 gene expression levels between tumor and normal groups. One-way ANOVA and Tukey’s Method were used to test significance ^*^p < 0.05, ^**^p < 0.01, ^***^p < 0.001, Abbreviation: ns: not significant.

Verification of gene expression levels is of great value in distinguishing cancer from the normal population. RT-PCR, as a common means to detect the expression level of gene mRNA, can show the transcription level of the gene. In this project, RT-PCR experiments were used to analyze the transcriptional differences of the IL18 gene between breast cancer and adjacent tissues. The histogram (Figure 10) showed that the IL18 was higher than that in adjacent tissues, and the results were consistent with the structure of the Bioinformatics Database, which provided evidence for the later functional experiments of the IL18. Raw results are shown in Supplementary Table 4.

## DISCUSSION

The correlation between altered metabolic reprogramming and tumor progression is confirmed by increasing studies. Lipid metabolism, as an important biological pathway, is involved in lipid uptake, lipogenesis, and oxidation in cells [23]. Cancer cells have been reported to take up energy through altered lipid metabolism pathways [24]. Dysregulation of lipid metabolism in tumor cells leads to the accumulation of free FAs, which increases the production of cellular ROS, thereby causing oxidative stress and promoting the secretion of cytokines in the tumor microenvironment [25, 26]. Meanwhile, more and more studies on lipid metabolism pathway genes have confirmed that they were involved in the regulation of tumorigenesis, development, and prognosis. For example, fatty acid-binding protein 4 (FABP4) has been confirmed to be a key factor in the provision of fatty acids by ovarian cancer cells [27]. In their sequencing investigation of breast cancer groups, Wang et al. [28] revealed that ACLY transcript levels in tumors were considerably greater than in normal tissues and positively linked with Ki67 expression. Lipid metabolism is crucial to tumor formation progression and mechanism response. However, previous studies have mainly focused on the functional study of single genes under specific factors and lacked systematic screening of prognostic indicators and mining of functional effects. Thus, we extensively examined breast cancer lipid metabolism-related genes to target immune function and find survival-predictive genes.

Relying on the data of breast cancer and paraneoplastic samples in the UCSC database, this study combined the gene sets of lipid metabolism and immune pathways in the GSEA and Immune databases for integration and screened the genes with bidirectional pathway effects. Firstly, we ran GO and KEGG functional enrichment analyses for the intersecting genes and discovered that they were mostly engaged in lipopolysaccharide, bacterial molecule, and steroid hormone biological processes. The KEGG results showed cytokine-enriched gene sets. KEGG found that the gene set was enriched in cytokine receptor interaction, the L-17 signaling pathway, neuroactive ligand-receptor interaction, and other pathways. According to KEGG, the gene set mainly responds to the immune response mechanism. Immediately after that, the model with risk score and survival prognosis was established by univariate Cox analysis and LASSO analysis, and nine genes with potential prognosis were filtered and screened out. The prognostic level of the high-risk group was worse than that of the low-risk group (*P* < 0.05), as shown by the K-M survival curve. The AUC of the ROC curve of the model was also about 0.7, which showed that the model’s prognostic features were accurate.

Demonstrate the expression levels and individual factor functions of the model gene set based on open-access databases. We mined the transcript level differences between breast cancer in situ tissues and normal tissues for nine genes based on the UALCAN database and also obtained the protein Log2 spectral count ratio values for three of the markers (CETP, IL18, and CXCL13) through the CPTAC database, and the histograms confirmed that the protein expression level differences with mRNA have consistency. In addition, immunohistochemistry results were mined for markers with protein data, and the results of staining of breast cancer tissues and normal breast tissue sections revealed significant differences in the expression levels of the target proteins (IL18 and CXCL13) in the real world. Simultaneous 3D structure prediction analysis for the nine proteins reveals that all nine indicators have complex structural features. The results of data analysis showed that the transcript and protein expression levels of CXCL13 and IL18 mRNA in the primary cancer tissues of patients with an early stage of breast cancer were highly consistent. The HE staining results of tissue sections also confirmed that the two genes had expression differences, and we hypothesized that IL18 and CXCL13 could be used as potential predictors to guide early clinical determination. Immediately after that, for the screening genes of the prognostic model, we mined the prognostic value of single gene expression levels for breast cancer through the GEPIA2 database. Five genes (CCL5, IL12B, IL24, IL18 and RORB) were screened with a significant correlation (*P* < 0.05) with overall survival (OS). Meanwhile, analyzing the single-gene ROC curves for the model, we found that the AUC values of some genes were > 0.7, among which the AUCs of CALR and IL18 were > 0.75 (*P* < 0.05). The results suggest that some single genes in the model not only have disease expression differences but also have strong prognostic guidance value, which can be used as potential clinical discriminators and prognostic indicators.

Increasing evidence confirms that lipid metabolism reprogramming occurs in malignant tumor cells, while metabolism-related genes have been reported to have drug-targeting functions. For example, the ATP citrate lyase (ACLY) gene inhibitor SB-204990 strongly inhibited tumor growth in lung, prostate, or ovarian cancer xenograft mice [29]. In contrast, intracellular FASN is a key lipogenic enzyme that catalyzes lipogenesis and mediates the extent of fibrosis [30, 31]. The FASN inhibitor TVB-2640 has been reported to undergo clinical trials targeting patients with solid tumors [32]. Based on this, we explored the correlation between small molecular compounds and candidate genes in tumor cell lines. First the CTRP database spearman correlation coefficients showed that all 9 prognostic genes possessed correlated small-molecule compounds; notably, among them, the expression level of IL18 had a positive correlation with the IC50 sensitivity of dozens of small-molecule compounds. Then, we mined the drug information of the candidate genes in the GSDC cell line database, the results of which similarly showed that the drug information of six of the candidate genes, among which FLT3 possessed a negative correlation with numerous small-molecule drugs, while the GSDC database similarly confirmed that the IL18 gene possessed positive synergistic effects with small-molecule compounds. Therefore, we hypothesized that FLT3 and IL18 genes in breast cancer patients may have important roles in targeting drugs, which can provide a basis for later pharmacodynamic evaluation and functional studies.

IL18, also known as interferon-γ-inducing factor) belongs to one of the members of the IL-1 superfamily [33]. IL18 has a variety of biological functions and has an important role in anti-inflammatory and anti-tumor immunity [34]. The latest study found that the IL-18 variant library screened for a special type of DR-18 that would not bind to decoys, and its experimental results showed significant efficacy when combined with PD-1 inhibitor treatment [35]. It was also confirmed that small-cell lung cancer secreted IL18, which enhanced the efficacy of CAR-T cell therapy targeting DLL3 [36]. The results of the study confirmed that IL18 has important prognostic value and a functional mechanism to regulate the immune response. Based on this, this project carried out a biological analysis of IL18 as the main research gene. Pan-cancer mRNA transcript level mining showed that IL18 has significant differences in its expression levels in a number of different types of malignant tumors. Additionally, DNA mutation information showed that IL18’s DNA mutation types are also different in these different types of tumors. Our subcellular localization results based on the HPA Breast Cancer Database showed that the IL18 response feedback occurred at the Golgi apparatus, Nucleoplasm, and Cytosol sites of cells. It is worth noting that the RT-PCR results also confirmed that the real-world IL18 expression level difference was consistent with the results of bioinformatics database. And the genetic down-regulation analysis of single-cell data from model animal mice pointed out that the IL18 gene was mainly expressed in stromal cells in normal breast tissues.

The expression pattern of IL18 indirectly indicates that this protein exercises important functional interaction mechanisms. In addition, we mined the OS analysis results of IL18 in pan-cancer by the K-M database, took the significance *P* < 0.05 as the filtering criterion, and filtered out a total of 5 malignant tumors with a correlation with the expression of IL18, except breast cancer. Meanwhile, combined with the information from TCGA patients’ survival data, it was clearly indicated that IL18 expression not only reduced the risk coefficients of BRCA, DLBC, and MESO but also increased the survival risk of patients with PAAD, LGG, and UVM. Meanwhile, for the functional study of IL18, it was found that the functional study of IL18 in breast cancer is still unclear, but past research reports pointed out that IL18 may be involved in the regulation of the uveal melanoma process, such as apoptosis, DNA repair, DNA damage, metastasis, and many other functional mechanisms. In summary, the IL18 gene not only has a screening value for cancer survival and prognosis, but its gene expression is involved in regulating multiple stages of cancer development.

After that, earlier research has shown that IL18 expression is linked to making cells more sensitive to drugs that weaken the immune system and to immune signaling responses to tumor immunosuppressants. For example, recent studies have indicated that DR-18, an isoform of IL18, enhances the efficacy of the immunosuppressant PD-1. In view of the close relationship between the lipid metabolism gene IL18 and immune mechanisms, we analyzed IL18 expression and the infiltration levels of T cell CD4+, T cell CD8+, neutrophil, DC cell, and macrophage by the TIMER2 algorithm, and the infiltration prognosis values indicated that IL18 expression might affect the T cell CD4+, neutrophil, and DC cell infiltration levels. We also mined the correlation of IL18 with immune subtypes of multiple cancers, in which cancer types with significant Kruskal-Wallis Test PV values were selected, e.g., IL18 was associated with C1 (wound healing), C2 (IFN-γ predominance), C3 (inflammation), C4 (lymphocyte depletion), and C6 (TGF-b predominance) in breast cancer. Meanwhile, we mined the immunosuppressant information related to IL18 in pan-cancer species and found that IL18 expression might be associated with breast cancer immunosuppressant (BTLA, CD244, CTLA4, CD96, etc.) drug sensitivity. Therefore, we hypothesized that IL18 expression not only affects the level of immune cell infiltration in the tumor microenvironment, but also has potential immunosuppressant synergistic and targeting effects.

In addition, this project mined the functional effects of the target gene, IL18, at the single-cell level. Several single-cell transcriptome data showed that IL18 was mainly localized in DC cells, as well as monocytes and macrophages. By integrating the set of genes positively and negatively regulated by IL18, GO analysis showed that IL18-related genes were mainly involved in adaptive immune response, and KEGG enrichment also showed that IL18 was mainly involved in adaptive immune response, lymphocyte activation involved immune response, and other functionalities. KEGG enrichment also showed that IL18 regulates adaptive immune response, lymphocyte activation involved immune response, and other multi-functions. The exact location of IL18 on a single cell level showed that its expression in breast cancer patients was mostly focused on the immune system. The functional enrichment of its protein interaction genes also showed that this gene changed the immune response in the tumor microenvironment.

## CONCLUSION

In this project, we estimated 136 genes that are involved in both lipid metabolism and immunology in breast cancer. And we developed a breast cancer characterization model that combines genes (CALR, CCL5, CXCL13, FLT3, IL12B, IL18) related to lipid metabolism and immune function. Meanwhile, the survival prognosis and functional mechanisms of the model gene as well as the key gene IL18, which has an important biological function in malignant tumors, were mined. This discovery can help improve the prognosis of breast cancer and the validation and development of drug therapy.

## Supplementary Materials

Supplementary Table 1

Supplementary Table 2

Supplementary Table 3

Supplementary Table 4
